# Prevention of Contrast-induced Nephropathy in Patients Undergoing Percutaneous Coronary Intervention

**DOI:** 10.2174/011573403X260319231016075216

**Published:** 2023-01-24

**Authors:** Raymond Pranata, Dendi Puji Wahyudi

**Affiliations:** 1 Department of Cardiology and Vascular Medicine, Faculty of Medicine, Universitas Padjadjaran, Hasan Sadikin General Hospital, Bandung, Indonesia

**Keywords:** Nephropathy, kidney, injury, prevention, percutaneous coronary intervention, adequate hydration, nephrotoxic medications

## Abstract

Contrast-induced nephropathy (CIN) or contrast-induced acute kidney injury has varying definitions, but in general, increased serum creatinine level by ≥ 0.3 mg/dL (26.5 µmol/L) or 1.5x of baseline value or urine output <0.5 mL/kg/h within 1-7 days after contrast media (CM) administration can be considered as CIN. CIN is one of the most common complications and is associated with increased mortality in patients undergoing percutaneous coronary intervention (PCI). Thus, risk stratification for CIN should be made and preventive strategies should be employed in which the intensity of the approach must be tailored to patient’s risk profile. In all patients, adequate hydration is required, nephrotoxic medications should be discontinued, and pre-procedural high-intensity statin is recommended. In patients with an estimated glomerular filtration rate (eGFR) <60 mL/min/1.73 m^2^, IV hydration should be started 12 hours pre-procedure up until 12-24 hours after the procedure. Remote ischemic preconditioning may be performed pre-procedurally. Radial first approach for vascular access is recommended. During the procedure, low or iso-osmolar CM should be used and its volume should be limited to eGFR x 3.7. In patients at high risk for CIN, additional contrast-sparing strategies may be applied, such as using a contrast reduction system, 5 Fr catheter with no sideholes, CM dilution, limiting test injection, confirming placement using guidewire, use of stent enhancing imaging technology, using metallic/software roadmap to guide PCI, use of IVUS or dextran-based OCT, and coronary aspiration. A more advanced hydration technique based on central venous pressure, left ventricular end-diastolic pressure, or using furosemide-matched hydration, might be considered.

## 1 INTRODUCTION

Contrast-induced nephropathy (CIN) or contrast-induced acute kidney injury has varying definitions (Table **1**) [1-4]. Kidney Disease Improving Global Outcome (KDIGO) defined it as serum creatinine level increase by ≥ 0.3 mg/dL (26.5 µmol/L) within 48 h or > 1.5-1.9 of the baseline value within 7 days or urine output <0.5 mL/kg/h after contrast media (CM) administration. European Society of Urogenital Radiology defined it as serum creatinine level increase by ≥ 0.5 mg/dL (44.2 mol/L) or > 25% of the baseline value 48-72 h after CM administration [4]. In general, increased serum creatinine level by ≥ 0.3 mg/dL (26.5 µmol/L) or 1.5x of baseline value or urine output <0.5 mL/kg/h within 1-7 days after CM administration can be considered as CIN.

CIN is one of the most common complications in patients undergoing percutaneous coronary intervention (PCI). An NCDR Cath-PCI registry showed the incidence of CIN as 7.1%, and 0.3% of the patients required dialysis [5]. In-hospital mortality was found to be significantly increased in patients with AKI (9.7%) and in those requiring dialysis (34%), compared to those without AKI (0.5%). AKI and dialysis were independent predictors of in-hospital mortality after multivariate adjustment. The incidence of AKI was 5.2% in eGFR >60 mL/min/1.73 m^2^, 8.0% in eGFR 45-60 mL/min/1.73 m^2^, 12.9% in eGFR 30-45 mL/min/1.73 m_2_, and 26.6% in eGFR <30 mL/min/1.73 m^2^. Another analysis derived from the NCDR Cath-PCI registry showed that patients with CIN had an increased risk of death, myocardial infarction, bleeding, and recurrent renal injury within one year after discharge [6].

## 2 PATHOPHYSIOLOGY

Iodinated contrast may cause direct injury through tubular toxicity property and reperfusion injury *via* free-radical mediated vasoconstriction due to vasospasm [7-9]. CM increases the viscosity of tubular fluid causing obstruction, and it may also induce the loss of polarity in tubular cells leading to apoptosis and necrosis, and increase sodium delivery to distal tubules, triggering tubuloglomerular feedback and subsequently causing renal vasoconstriction. Disturbances in renal blood flow cause arteriolar vasoconstriction and increased blood osmolality and viscosity. Consequently, this condition induces ischemia and microvascular thrombosis, and impairs renal function [10].

## 3 RISK FACTORS

The presence of pre-existing renal disease is the main risk factor for CIN, exhibiting a non-linear relationship [5]. Diabetes is also a risk factor for CIN, regardless of the concomitant nephropathy. Elevated pre-procedural glucose levels are associated with the risk of CIN, which might be mediated by endothelial dysfunction, increased activation of prothrombotic factors, vascular inflammation, and reactive oxygen species production [11]. Elderly individuals may have a decline in renal function, characterized by a reduction in GFR, tubular secretion, and the ability to control urine. They may also have difficult vascular access and more complex coronary artery disease, necessitating more complex intervention, thus increasing the need for CM use [12]. The presence of anemia has the potential to contribute to renal ischemia, consequently leading to susceptibility to CIN. A study showed the risk of CIN to be higher in patients with lower baseline hematocrit. A study showed that for every 3% decrease in baseline hematocrit, the risk of CIN increased by 11% in patients with chronic kidney disease (CKD) and 23% in patients without CKD. Multivariate analysis showed that a decrease in hematocrit was independent of the presence of CKD [13]. Congestive heart failure (CHF), reduced left ventricle systolic function, dehydration, hypotension, and intra-aortic balloon pump use were associated with increased CIN risk [12]. Patients with CHF show an increased risk for hemodynamic instability, which may lead to renal hypoperfusion. Renal congestion and poor systolic function lead to deterioration of renal function. The utilization of an intra-aortic balloon pump may indicate the presence of hemodynamic instability, necessitating further hemodynamic support. Moreover, the utilization of an intra-aortic balloon pump has been associated with atheroembolism and the partial obstruction of renal blood flow [12].

The Mehran Risk Score is widely used to provide risk stratification for CIN and dialysis in patients undergoing PCI [14]. The risk score is based on risk factors, including hypotension, use of intra-aortic balloon pump, CHF, age >75 years, anemia, diabetes, CM volume, serum creatinine, or eGFR. This score has also been validated in ST-segment elevation myocardial infarction (STEMI) undergoing primary PCI [15]. The risk score should not be used in patients with end-stage renal disease on dialysis and patients with contrast exposure within 1 week of the index procedure.

## 4 CONTRAST MEDIA

Nonionic, low-osmolar contrast media (LOCM) should be used for most patients and iso-osmolar contrast (IOCM) agents can be considered in patients with chronic kidney disease, although studies have shown no benefit of iso-osmolar compared to low-osmolar contrast [16]. A meta-analysis of 10 studies demonstrated IOCM *vs.* LOCM in patients with CKD stage 3 and above undergoing coronary angiography to not be associated with a reduction in CIN [OR=0.72 (CI: 0.50-1.04), *p*=0.0^8^] [17].

The amount of CM use correlates with the risk of CIN; thus, it is important to restrict CM utilization. SCAI expert consensus statement: 2016 best practices in the cardiac catheterization laboratory and ESC/EACTS 2018 Guideline on Myocardial Revascularization recommend a maximum CM limit of eGFR x 3.7 per procedure [16, 18]. This is based on the ratio of contrast volume to creatinine clearance ratio of 3.7 that has been found to be predictive of CIN (OR of 3.84, 95% CI 2.0 to 7.3; *p* < 0.001) after adjustment [19].

## 5 PRE-PROCEDURAL MEDICATIONS

Various pre-procedural medications have been investigated to reduce the risk of CIN. However, most of the studies have shown a high risk of bias and small-study effects upon the pooling of studies. Thus, only statin is currently recommended in the guidelines [20].

Rosuvastatin has been shown to reduce the incidence of CIN. The PRATO-ACS trial demonstrated that patients with non-ST elevation acute coronary syndrome (ACS) who were administered 40 mg rosuvastatin at admission, followed by 20 mg daily, had a reduced incidence of CIN compared to those who did not receive any statin treatment (OR 0.38; 95% CI 0.20-0.71, *p*=0.003) [21]. TRACK-D study involving 2998 patients with type 2 diabetes mellitus and CKD undergoing coronary or peripheral arterial angiography with or without PCI showed stat rosuvastatin 10 mg daily to reduce CIN incidence (2.3% *vs.* 3.9%, *p*=0.01) compared to standard-of-care [22]. Additionally, PRATO-ACS and TRACK-D studies showed reduced incidence of worsening heart failure and adverse cardiovascular and renal events, such as death, dialysis, myocardial infarction, stroke, or persistent renal damage. Currently, the 2021 ACC/AHA/SCAI Guideline for Coronary Artery Revascularization suggests pre-treatment with high-intensity statins [20]. ESC/EACTS 2018 Guideline on Myocardial Revascularization recommends rosuvastatin 40/20 mg or atorvastatin 80 mg for the prevention of CIN [18].

The acetylcysteine for contrast-induced nephropathy trial (ACT) showed that in 2308 patients undergoing an intravascular angiographic procedure with at least one risk factor for contrast-induced acute kidney injury, the incidence of CIN was similar in both n-acetylcysteine and control groups (12.7% and 12.7%) [23]. The combined endpoint of death and dialysis was similar in both the groups. PRESERVE trial showed that N-acetylcysteine 1200 mg 1 hour before angiography and 1 hour after angiography in 5177 patients with high risk for renal complications, continued twice daily for four days, showed no benefit of IV sodium bicarbonate over IV sodium chloride or oral N-acetylcysteine over placebo for the prevention of death, need for dialysis, or a persistent decline in kidney function at 90 days or for the prevention of CIN [24]. Currently, the 2021 ACC/AHA/SCAI Guideline for Coronary Artery Revascularization suggests to not administer N-acetyl-L-cysteine to prevent CI-AKI [20].

A meta-analysis of 7 randomized controlled trials showed nicorandil as associated with a reduction in CIN [OR 0.31 (0.20, 0.46), *p* < 0.00^1^] [25]. A study conducted by Ko *et al.* revealed a non-significant effect of nicorandil administration; however, it is worth noting that this study was the earliest and the sole investigation that administered intravenous nicorandil. Other studies have shown a statistically significant reduction in CIN. A meta-analysis concluded nicorandil to have moderate certainty of evidence; however, it is important to note that all the studies included in the study were conducted in Asia. Therefore, additional research is required to validate whether these findings can be generalized to other populations. Trimetazidine was shown to significantly reduce the incidence of CIN in a pooled meta-analysis of 7 studies [RR 0.46 (0.34, 0.63), *p*<0.00^1^] [26].

Discontinuation of metformin and nephrotoxic drugs has also been recommended, while discontinuation of renin-angiotensin-aldosterone inhibitors remains controversial [27, 28].

## 6 HYDRATION

Hydration dilutes contrast concentration in the glomerular filtrate and reduces oxygen consumption due to less sodium reabsorption. Intravenous hydration should be administered in moderate and high-risk patients based on the Mehran risk score. An intravenous pre-hydration should be given using NaCl 0.9% for 1 mL/kg/h for 12 hours (or 3 mL/kg for 1 hour) and continued post-procedurally using NaCl 0.9% for 1 mL/kg/h for 12-24 hours (0.5 mL/kg/h if LVEF ≤35% or NYHA >II) [18, 28]. The fluid must be preferably normal saline because no advantages have been shown by other solutions [27].

In a study by Qian *et al*., central venous pressure (CVP)-guided fluid administration reduced CIN incidence (15.9% *vs.* 29.5%; *p* =0.006), and CVP-guided fluid administration resulted in higher fluid administration (1,827±497 ml *vs.* 1,202±247 ml; *p* < 0.001) without increased acute heart failure incidence (3.8% *vs.* 3.0%; *p* = 0.500). A subgroup analysis showed LVEF <40% to provide more benefit (17.5% *vs.* 33.3%; *p* = 0.031), and the patients with CVP level <6 cmH2O received the greatest benefit from the CVP-guided hydration (10.7% *vs.* 37.5%; *p* = 0.045) [29].

POSEIDON randomized controlled trial evaluated fluid administration based on LV-end diastolic pressure (LVEDP), where CIN occurred less frequently (6.7%) in patients subjected to LVEDP-guided hydration compared to the control group (16.3%) (RR 0.41, 95%CI 0.22–0.79; *p*=0.005). In this study, bolus infusion at 3 mL/kg for 1 h was given before the procedure. LVEDP-guided protocol followed 5 mL/kg/h for LVEDP <13 mmHg, 3 mL/kg/h for LVEDP of 13–18 mm Hg, and 1.5 mL/kg/h for LVEDP >18 mmHg. The control group received 1.5 mL/kg/h fluid administration. The fluid rate was set at the start of the procedure, continued intra-procedure, and for 4 h post-procedure.

Furosemide with matched hydration (FMH) using RenalGuard aimed to produce high urine output and fluid balancing, which diluted CM and increased its excretion. This was achieved by 250 ml IV bolus of NaCl 0.9%, followed by IV furosemide bolus (0.5 mg/kg); the infusion was given precisely and adjusted based on the patient’s urine output. The device measures the patient's urine output and infuses NaCl 0.9% to balance the fluid status, thus enabling rapid removal of CM while also maintaining fluid balance. The coronary procedure was started once the urine output was >300 mL/h. In the matched hydration compared to standard hydration for contrast-induced nephropathy prevention (MYTHOS) trial, the incidence of CIN was lower in the FMH group at 4.6% *vs.* 18%, *p*=0.005 [29]. The incidence of in-hospital complications was also lower in the FMH group at 8% *vs.* 18%. Renal insufficiency after contrast media administration trial II (REMEDIAL II) showed that FMH reduced CIN compared to the sodium carbonate + N-acetylcysteine group (11% *vs.* 20.5%; OR, 0.47; 95% CI 0.24-0.92) [30]. This method resulted in high urine flow, speeding up the release of contrast through the tubules, diluting the concentration and contact time of contrast, and also reducing the oxygen demand of the kidney.

## 7 REMOTE ISCHEMIC PRECONDITIONING

Remote ischemic preconditioning can be performed by inflating blood pressure cuffs at the patient’s limb for three or four cycles of 5 minutes of inflation (ischemia) and 5 minutes of deflation (reperfusion). This exerts an ischemia-protecting effect on the renal medulla by reducing the reperfusion injury [7-9]. Ischemia protection has been reported to be achieved through the reperfusion injury salvage kinase (RISK) pathway, hypoxia-inducible factor system, and endogenous antioxidant system, suppressing the induction of renal inflammation and preventing renal tubular cell apoptosis [31]. Neuro-humoral substances, such as opioids, micro-RNA-144, bradykinin, erythropoietin, prostaglandins, nitric oxide, O-linked β-N-acetylglucosamine, and adenosine are also activated through ischemic preconditioning [31, 32]. A meta-analysis showed the reduced incidence of CIN (OR 0.41 (95% CI 0.29-0.58, *p*<0.001) in patients undergoing PCI [33]. Time ≤45 minutes, ≤60 minutes, and ≤120 minutes before the procedure, and 4 cycles or 3 cycles protocol have similar effectiveness in terms of CIN reduction. Three cycles are preferable in STEMI and other patients that require immediate intervention.

## 8 PROCEDURAL STRATEGIES

Procedural strategies to reduce the risk of CIN revolve mainly around contrast-sparing strategies and reducing CM delivery to systemic circulation.

### 8.1 Radial Access

The AKI-MATRIX trial showed that radial access resulted in less AKI compared to femoral access (15.4% *vs.* 17.4%, OR 0.87; 95%CI 0.77-0.98; *p* = 0.0181). The benefit was more pronounced in patients at greater risk for AKI [34]. Radial access is associated with less bleeding risk, thus reducing the risk of hemodynamic instability and renal ischemia. Using radial access also reduces the manipulation of the abdominal aorta.

### 8.2 Biplane Angiography

The RAMBO trial evaluated biplane *versus* monoplane fluoroscopy in patients undergoing PCI [35]. Biplane angiography was associated with lower contrast use (92 mL *vs.* 108 mL, *p*<0.001), but higher radiation exposure at the left arm (4 *vs.* 2 uSv, *p*<0.001) and higher dose area product (11,955 *vs.* 8,349 mGy*cm^2^, *p*<0.001) without difference in fluoroscopy time (4.4 min *vs.* 4.3 min, *p*=0.89). Thus biplane fluoroscopy can be considered in patients with a high risk of CIN, but the default approach should be monoplane fluoroscopy.

### 8.3 DyeVert System

The DyeVert™ contrast reduction system (Osprey Medical Inc., Minnetonka, MN, USA) and the next-generation DyeVert™ Plus Contrast Reduction System (Plus System, OsPrey Medical) provide pathway resistance to the fluid using a dedicated diversion valve. The diversion valve self-adjusts to the manual injection pressure to divert CM into the reservoir chamber within the module. This excess CM volume transferred to the reservoir is thought to contribute to reflux to the aortic root, rather than to opacification of coronary arteries. This diverted volume of CM does not enter the patient. The DyeVert™ contrast reduction system (Osprey Medical Inc., Minnetonka, MN, USA) leads to a 41.0% reduction in CM volume (36.9±10.9 mL *versus* 62.5±12.7mL, *p*<0.001) with non-inferior image quality (*p*=0.03) [36]. A multicenter study on patients with eGFR 20-60 mL/min/1.73 m^2^ showed that the use of DyeVert™ Plus Contrast Reduction System (Plus System, OsPrey Medical) resulted in CM volume savings of 40.1 ± 8.8% (95%CI 38.4 - 41.8; *p*< 0.0001) per procedure [37]. Image quality was maintained in all but one case in which the system was turned off for one injection.

### 8.4 Angiography and PCI Techniques

In order to enhance the contrast use efficiency, the utilization of 5-Fr catheters without side-holes for CM injection can be employed [38]. Additionally, diluting CM with 50% NaCl 0.9% reduced the total CM administered without compromising image quality [39]. To achieve a further decrease in contrast media (CM) administration, it is recommended to restrict the volume of CM each injection to 2 mL. The operator can also reduce test injections, and instead of injecting CM, the operator may confirm guiding catheter engagement by a guidewire, and if the operator encounters difficulties during side branch wiring, intravascular ultrasound in live-view mode can be used to guide wiring [38]. Displaying videos of previous coronary angiography side-by-side with the current fluoroscopy can reduce the need for acquiring new images. Stent enhancement techniques (*e.g.*, StentBoost, Philips and ClearStent, Siemens) can provide clear visualization of the stent; thus, these can be useful post-PCI to reduce cine angiography. To avoid continuous injection of CM during wiring and coronary intervention, the operator can use additional guidewires to create a metallic roadmap of target vessels and their branches; alternatively, software, such as Dynamic 3D Roadmaps and Philips, can be used to guide PCI [38].

### 8.5 IVUS and Dextran-based OCT

The MOZART study evaluated intravascular ultrasound (IVUS)-guided PCI to minimize contrast use. Non-contrast fluoroscopy and cine may be used to visualize the stent limits and borders, identify the IVUS probe position inside the vessel, and register balloon expansion. Baseline IVUS determines whether lesion preparation is required or direct stenting can be performed. Pre-dilation is evaluated using IVUS rather than angiography and stent sizing is based on IVUS. Manual IVUS imaging is conducted to precisely identify the proximal and distal reference spots. IVUS is used to determine whether subsequent dilatation or stenting is needed. The median CM volume in the angiography group in the study has been found to be 64.5 ml and 20.0 ml in the IVUS-guided group (*p* < 0.001) [39]. Thus, this technique is feasible and should be considered in patients at high risk for CIN.

Optical coherence tomography (OCT) can provide a high-resolution image and distinguish between lipid-rich plaques, fibrous plaques, calcium, and thrombus in a lesion. Like IVUS, OCT can be used to guide PCI and minimize angiographic acquisition. However, OCT requires red blood cells’ displacement, and usually, CM is used for this purpose. Low-molecular-weight dextran has been proposed to replace CM, and a study showed that in 3418 cross-sections (1,709 with contrast and 1,709 with dextran), clear image segments of cross-sections were similar in contrast and dextran groups (97.0% *vs.* 96.7%, *p*=0.45). With similar mean lumen areas (correlation coefficient of 0.984) [40], there was no complication related to OCT imaging in both the contrast and dextran groups. Thus low-molecular weight dextran may be used as a CM alternative for OCT imaging in patients at high risk for CIN.

### 8.6 Coronary Sinus Aspiration

Coronary sinus aspiration is performed by cannulating the coronary sinus *via* the subclavian or femoral vein, and then aspiration is performed through the transseptal sheath or balloon occlusion catheter (determined based on coronary sinus caliber compared to the sheath) simultaneously during each coronary injection, in which fluoroscopy is continued until appearance of contrast in CS and the clearance by aspiration. In a study, the mean fraction of aspirated contrast has been found to be 39.35±10.47% in the coronary sinus aspiration group. The incidence of CIN was reduced in the coronary sinus aspiration group (5.55% *vs.* 36%, *p=*0.028) and there was no significant difference between the post-procedural Hb in both groups (*p*=0.06). Coronary sinus cannulation time was 19.27±3.54 minutes and time to clearance of contrast was 11.3±0.73 seconds. The technique is cumbersome, but it may be considered in patients at very high risk of developing CIN. The fluoroscopy time is also prolonged due to the waiting time until the contrast is fully cleared.

## CONCLUSION

Risk stratification for CIN should be performed in patients who are planned for coronary angiography or PCI. Following risk stratification, preventive measures should be implemented (Table **2**), particularly in individuals deemed to be at high risk for developing CIN for whom a more intensive approach is warranted. In all patients, adequate hydration is required, nephrotoxic medications should be discontinued, and pre-procedural high-intensity statin is recommended. In patients with eGFR <60 mL/min/1.73 m^2^, IV hydration should be started 12 hours before the procedure up until 12-24 hours after the procedure. Remote ischemic preconditioning may be performed pre-procedurally. Radial first approach for vascular access is recommended. Intra-procedure, low or iso-osmolar CM should be used, and its volume should be limited to eGFR x 3.7. In patients at high risk for CIN, additional contrast-sparing strategies may be applied, such as DyeVert™ contrast reduction system, 5 Fr catheter with no sideholes, CM dilution, limiting test injection, confirming placement using guidewire, use of stent enhancing imaging technology, using metallic/software roadmap to guide PCI, use of IVUS or dextran-based OCT, and coronary aspiration. A more advanced hydration technique based on CVP, LVEDP, or using RenalGuard™, might be considered. Although most studies on CIN prevention have not investigated long-term MACE, it is expected that by preventing CIN and dialysis, MACE can also be reduced. Moreover, several measures listed may have cardioprotective mechanisms, for example, statin. Thus, exhaustive steps should be taken to prevent CIN.

## Figures and Tables

**Table 1:** Definition of contrast-induced nephropathy.

**-**	**KDIGO [** **1** **]**	**AKIN [** **2** **]**	**ADQI [** **3** **]**	**ESUR[** **4** **]**
SCr (mg/dL)	≥ 0.3 within 48 hours	≥ 0.3 within 48 hours	-	≥ 0.5 within 48-72 hours
SCr (multiplier)	1.5-1.9x within past 7 days	1.5-2.0x	≥ 1.5x over 1-7 days, sustained for >24 hours	≥ 1.25x within 48-72 hours
eGFR	-	-	Decrease ≥25% over 1-7 days, sustained for >24 hours	-
Urine Output (mg/kg/h)	<0.5 for 6-12 hours	<0.5 for >6 hours	<0.5 for 6-12 hours	-

**Abbreviations:** SCr: Serum Creatinine; eGFR: estimated Glomerular Filtration Rate; KDIGO: Kidney Disease: Improving Global Outcomes; AKIN: Acute Kidney Injury Report; ADQI: Acute Dialysis Quality Initiative; ESUR: European Society of Urogenital Radiology.

**Table 2:** Strategies to prevent contrast-induced nephropathy

**Pre-Procedural**	**Intra-Procedure**	**Post-Procedural**
NaCl 0.9% for 1 mL/kg/h for 12 hours before procedure (0.5 mL/kg/h if LVEF ≤35% or NYHA >II)	Displaying videos of previous coronary angiography to reduce the need for acquiring new images	NaCl 0.9% for 1 mL/kg/h for 12-24 hours after procedure (0.5 mL/kg/h if LVEF ≤35% or NYHA >II)
Discontinue metformin and nephrotoxic drugs	Biplane angiography	Continue high-intensity statin
High-intensity statin	Low or iso-osmolar contrast, Dilute contrast by 50%	-
Remote ischemic pre-conditioning	Radial Access	
-	Use 5-Fr catheters with no side-holes	
	Hydration using RenalGuard^TM^, LVEDP, or CVP guided	
	Limit contrast use < eGFR x 3.7	
	Limiting CM volume per injection (2 mL/injection)	
	Aspirate contrast from guiding catheter before introducing equipment	
	Reduce test injection, use guidewire or IVUS instead	
	Use DyeVert^TM^ Contrast Reduction system	
	Use stent enhancement technology	
	Use metallic/software roadmap to map vessels	
	IVUS or dextran-based OCT guided PCI	
	Coronary Sinus Aspiration	

**Abbreviations:** CM: contrast media; eGFR: estimated glomerular filtration rate; CVP: central venous pressure; LVEDP: left ventricular end-diastolic pressure; LVEF: left ventricular ejection fraction; IVUS: intravascular ultrasound; OCT: optical coherence tomography; PCI: percutaneous coronary intervention; NYHA: New York Heart Association

## LIST OF ABBREVIATIONS

CIN: Contrast-induced Nephropathy
CM: Contrast Media
PCI: Percutaneous Coronary Intervention
CKD: Chronic Kidney Disease
CHF: Congestive Heart Failure
